# N-CAM Exhibits a Regulatory Function in Pathological Angiogenesis in Oxygen Induced Retinopathy

**DOI:** 10.1371/journal.pone.0026026

**Published:** 2011-10-17

**Authors:** Joakim Håkansson, Anders Ståhlberg, Fredrik Wolfhagen Sand, Holger Gerhardt, Henrik Semb

**Affiliations:** 1 Department of Medical Biochemistry, Sahlgrenska Academy at Gothenburg University, Göteborg, Sweden; 2 Department of Pathology, Sahlgrenska Cancer Center, The Sahlgrenska Academy at University of Gothenburg, Göteborg, Sweden; 3 TATAA Biocenter, Göteborg, Sweden; 4 Stem Cell and Pancreas Developmental Biology, Department of Laboratory Medicine, Stem Cell Center, Lund University, Lund, Sweden; 5 Vascular Biology Laboratory, London Research Institute-Cancer Research UK, London, United Kingdom; Center for Regenerative Therapies Dresden, Germany

## Abstract

**Background:**

Diabetic retinopathy and retinopathy of prematurity are diseases caused by pathological angiogenesis in the retina as a consequence of local hypoxia. The underlying mechanism for epiretinal neovascularization (tuft formation), which contributes to blindness, has yet to be identified. Neural cell adhesion molecule (N-CAM) is expressed by Müller cells and astrocytes, which are in close contact with the retinal vasculature, during normal developmental angiogenesis.

**Methodology/Principal Findings:**

Notably, during oxygen induced retinopathy (OIR) N-CAM accumulated on astrocytes surrounding the epiretinal tufts. Here, we show that N-CAM ablation results in reduced vascular tuft formation due to reduced endothelial cell proliferation despite an elevation in VEGFA mRNA expression, whereas retinal developmental angiogenesis was unaffected.

**Conclusion/Significance:**

We conclude that N-CAM exhibits a regulatory function in pathological angiogenesis in OIR. This is a novel finding that can be of clinical relevance in diseases associated with proliferative vasculopathy.

## Introduction

Proliferative vascular malformations are the cause of sight threatening complications in diseases such as diabetic retinopathy, the dominant cause of blindness in people <60 in developed countries [1], and retinopathy of prematurity (ROP), the primary cause of blindness in infancy [2]. Common to these complications, initial retinal ischemia is considered to trigger a series of events including epiretinal neovascularization, vitreous hemorrhages and traction retinal detachment, which eventually lead to blindness. The oxygen induced retinopathy (OIR) model mimics blood vessel pathologies and has been used to study proliferative retinopathy in mice [3]. In OIR, mice at postnatal day seven (P7) are exposed to hyperoxia (75% O_2_) causing the retinal vasculature to regress centrally. When returning the mice to normal oxygen level at P12, the local hypoxia within the capillary free zone induces revascularization and pathological intravitreous neovascularization in the form of epiretinal tuft formation [3], [4], [5].

Vascular endothelial growth factor A (VEGFA) is a potent mitogen and chemoattractant for endothelial cells [6], [7], [8]. VEGFA induces retinal blood vessel development both under normal conditions [4] and in pathological proliferative retinopathies [5]. During development of the retinal vasculature, VEGFA is expressed in two layers of the retina in response to local hypoxia [4]. After birth, the superficial blood vessel layer develops peripherally from the optic disc. Astrocytes in the ganglion cell layer express VEGFA to support endothelial tip cell guidance and migration [9]. As the retina thickness increases during the first week, hypoxic conditions stimulate VEGFA expression by cells in the inner nuclear layer (presumably Müller cells) [4], leading to development of the deep blood vessel plexus. Two weeks after birth the retinal vasculature is largely completed. Crucial for vasculature formation is VEGFA binding to the extra cellular matrix (ECM) to build up a gradient around the migrating endothelial tip cells [9]. This gradient guides the tip cells which are probing their way with filopodia extensions. Disruption of this gradient leads to disturbed guidance and defective vascular development [9]. Although both normal and pathological retinal angiogenesis stems from local ischemia, there is an essential difference in the direction of blood vessel growth. In the former, the blood vessels are first restricted to the superficial ganglion cell layer after which they infiltrate the deeper retinal layers. However, during pathological angiogenesis blood vessels penetrate the exceeding inner limiting membrane to form intravitreous epiretinal tufts [3], [4], [5]. Importantly, the mechanism behind formation of epiretinal tufts remains unknown, whereas revascularization of the avascular area proceeds as in normal blood vessel development.

The neural cell adhesion molecule (N-CAM) is expressed throughout the retina and has pronounced expression in Müller cells and astrocytes [10], [11], [12]. Müller cells have been reported to be in close contact with the vasculature, producing matrix molecules and having important functions in regulating signal transduction in the retina [11], [13], [14], [15], [16]. Astrocytes form the pre-existing cell layer on which the superficial blood vessel plexus develops [17], [18]. Recently, we showed that N-CAM regulates pathological angiogenesis during tumor progression [19], suggesting that N-CAM may also be involved in pathologic angiogenesis of proliferative retinopathy. To address whether N-CAM plays such a role, we analyzed the consequence of N-CAM ablation on OIR. Here, we show that during OIR, N-CAM accumulates in astrocytes closely associated with the tufts. Furthermore, N-CAM-deficiency resulted in reduced vascular tuft formation and endothelial cell proliferation, despite an elevation in VEGFA mRNA expression.

## Results

### N-CAM ablation does not perturb normal development of the retinal vasculature

**Figure 1:**
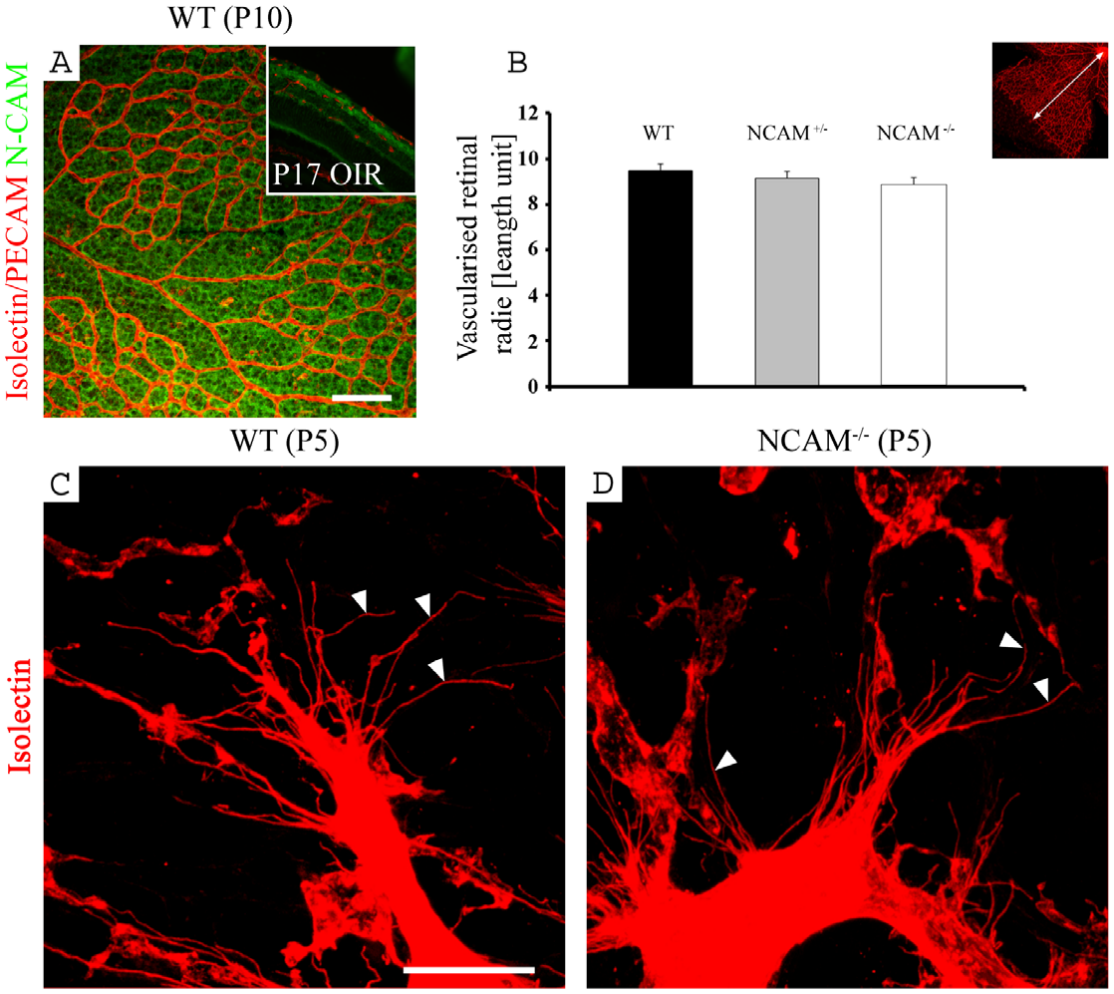
N-CAM is expressed in the mouse retinal blood vessel layers and N CAM deficiency does not affect the radial blood vessel development. (A–D) Retinal blood vessels visualized by isolectin staining (red). (A) Confocal image of a wt P10 retina, additionally immunostained with an antibody against N-CAM (green). As inset in (A) a section from a P17 OIR retina is shown, stained with N-CAM (green) and PECAM (red). The N-CAM expression is evenly distributed radially over the retina. (B) Statistic analysis of the radial development of the blood vessel plexus at P5 shows that there is no difference in normal blood vessel development between N-CAM deficient mice and wt controls. The inset picture illustrates the radial blood vessel development at P5. Statistical method used was Student t-test. (n_wt_ = 15, n_N-CAM+/−_ = 19, n_N-CAM−/−_ = 16). (C, D). Confocal images of sprouting tip cells in the border of the developing vasculature at P5 show that the sprouting in the N-CAM deficient retina (D) is identical to the wt (C). Arrowheads indicate filopodia which are stained by isolectin. Scale bars are 100 µm (A), and 50 µm (C).

To examine whether N-CAM is required for developmental angiogenesis in the retina, we studied normal development of the retinal vasculature in N-CAM-deficient mice. At P10, when the superficial blood vessel plexus is almost fully developed, N-CAM expression was localized in the vascular region of the ganglion cell layer in wild type (wt) mice (Figure 1A). At P7, N-CAM expression was localized around the GFAP^+^ astrocytes in close contact with the vasculature, but even more pronounced in the deeper layers of the retina (Figure S1A). Ablation of N-CAM resulted in no effect on radial development or tip cell sprouting, (Figure 1B–D), and there was no significant difference between wt and N-CAM^−/−^ when comparing the vessel diameter in the capillary network in P10 retinas (wt: 7.5±0.3, N-CAM^−/−^: 8.0±0.2, n_both groups_ = 3.

### Epiretinal tufts are surrounded by N-CAM expressing astrocytes

**Figure 2:**
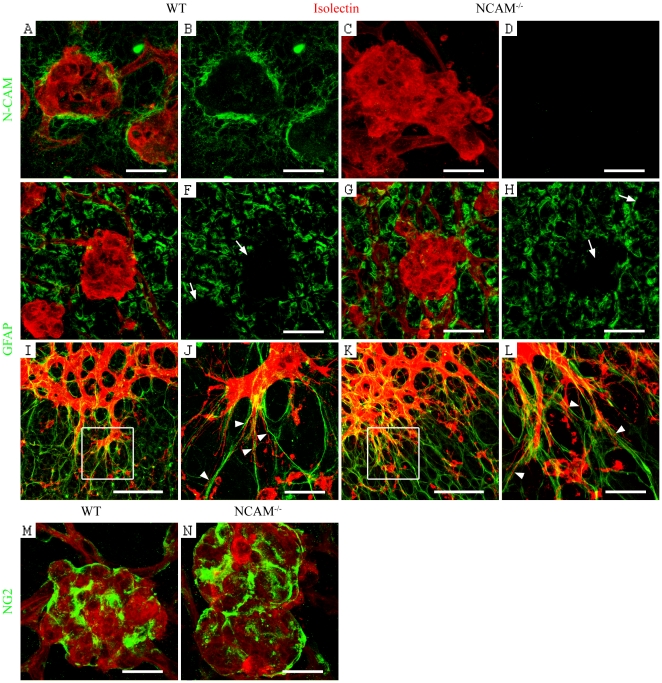
N-CAM is expressed by astrocytes around the epiretinal tufts. (A–N) Confocal images of retinal blood vessels, visualized by isolectin (red) and additionally stained with antibodies against N-CAM (A–D), GFAP (E–L) and NG2 (M, N) (green). N-CAM is accumulated around the epiretinal tufts (A, B), probably expressed by the surrounding GFAP^+^ astrocytes (E, H). The tufts give rise to empty (GFAP^−^) spots in the astrocyte layer (indicated by arrows in F and H). (C, D) Negative controls of N-CAM expression in N-CAM deficient retinas. (I, K) Overview pictures of the vascular front at P5. The marked square is enlarged in (J, L), arrowheads mark the filopodia attachment to the GFAP^+^ astrocytes. The astrocyte layer appeared identical in wt (E, F, I, J) and N-CAM deficient retinas (G, H, K, L). (M, N) NG2^+^ pericytes cover the tufts in both wt (M) and N-CAM deficient (N) retinas. Scale bars are 20 µm (A–D, J, L, M, N), 40 µm (E–H) and 50 µm (I, K).

Notably, N-CAM, which normally is distributed between blood vessels on astrocytes (S1A), was accumulated around the retinal tufts during OIR (Figure 2A–D). In OIR, the tufts are surrounded by astrocytes and can be visualized as GFAP^−^ spots in areas of tuft formation (Figure 2E–H). During blood vessel development in the retina, the blood vessels send out thin filopodia along the pre-existing astrocyte network. This process is not disturbed in the N-CAM deficient mice (Figure 2I–L). In OIR the other cell type in contact with the tufts are pericytes, but since they cover the entire surface of the tuft (Figure 2M, N) and N-CAM distribution was limited to the edges, matching the astrocyte location, the probable source for N-CAM is astrocytes.

### N-CAM ablation decreased pathological angiogenesis without affecting the avascular area in OIR

**Figure 3:**
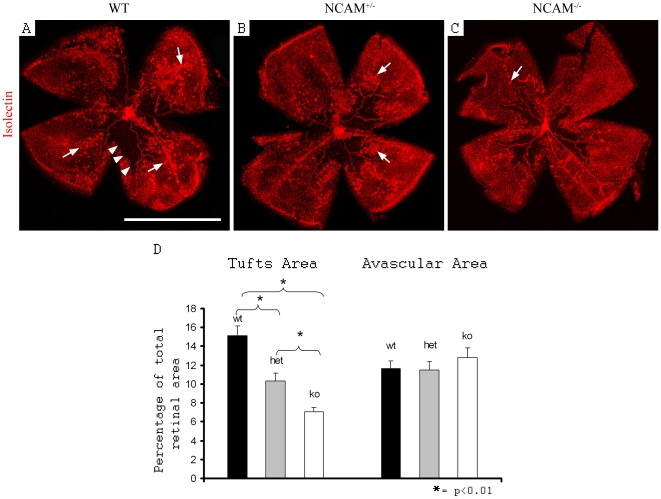
N-CAM deficiency leads to decreased pathological angiogenesis but unaffected avascular area in OIR. (A–C) Retinal blood vessels visualized by isolectin staining illustrating the tuft formation (arrows) and avascular area (arrowheads) at P17 in OIR in wt (A), heterozygous- (B) and homozygous (C) N-CAM mutated retinas. (D) Statistic analysis of tuft formation and avascular area showing a linear decrease in tuft formation from wt to homozygous N-CAM deletion, but unaffected avascular area. Statistical method used was Student t-test (n_wt_ = 15, n_N-CAM+/−_ = 19, n_N-CAM−/−_ = 16). * = p<0.05. Scale bar is 1 cm.

To examine whether N-CAM affected pathological angiogenesis, including retinal revascularization and tuft formation, during OIR, we analyzed N-CAM-deficient mice after five days in normoxia (P17). Heterozygous and homozygous N-CAM mutant animals exhibited a gene-dosage dependent reduction of tuft formation (Figure 3A–D). The number of tufts was markedly reduced in N-CAM knock outs compared to wt (Figure S2A). Also by plotting the distribution of tufts in a size dependent manner, it can be seen that there were more tufts of all sizes in wt compared to N-CAM homozygous knock outs (Figure S2B). However, there was no difference in the size of the avascular area compared to wt littermates (Figure 3D).

### N-CAM ablation does not affect blood vessel leakage

We have earlier reported that N-CAM ablation leads to increased leakage on tumor blood vessel [19]. To analyze whether N-CAM has the same effect on the vascular integrity in pathological angiogenesis during OIR the vasculature of mice after OIR was perfused with FITC-labeled dextran. The perfusion revealed no difference in blood vessel leakage in the N-CAM mutants compared to wt (Figure S1B).

### Despite increased VEGFA level in N-CAM deficient retinas, the proliferation rate in tuft endothelium was decreased

**Figure 4:**
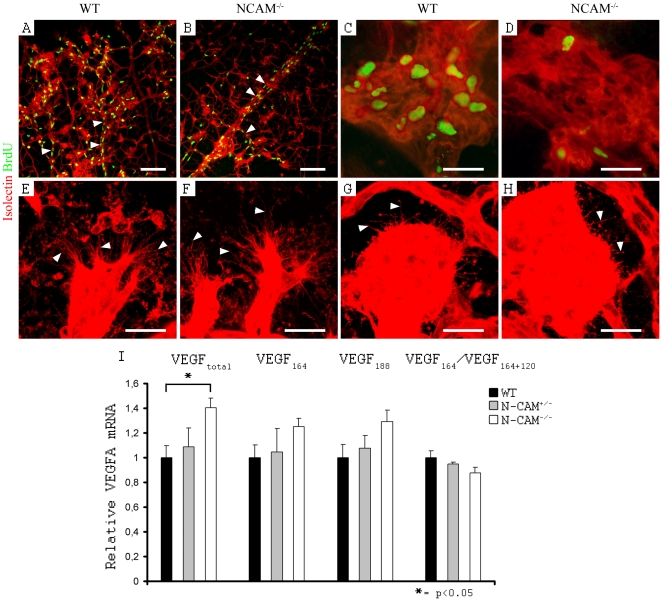
Decreased proliferation in tuft endothelium despite increased VEGFA level in N-CAM deficient retinas in OIR. (A–H) Confocal images of retinal blood vessels, visualized by isolectin (red). (A–D) Additional antibody staining against BrdU to view the proliferating nuclei (green). (A, B) Decrease in endothelial cell proliferation in N-CAM deficient retinas (B) compared to wt (A) was restricted to the tufts, whereas the remaining vasculature was unaffected (arrowheads indicate veins). (C, D) High magnification of an epiretinal tuft with a clear down regulation of endothelium proliferation in N-CAM deficient retina (D) compared to wt (C). (E–H) Endothelial sprouting in the revascularization of the avascular area (E, F), and in the tufts (G, H), after OIR treatment. There was no change in sprouting in N-CAM deficient mice (F, H) compared to wt (E, G). Arrowheads indicate the sprouts. (I) Graphic illustration of mRNA quantification by QPCR of VEGFA levels in retinas at P17 after OIR treatment. N-CAM deficient mice exhibited a 40% increase in total VEGFA mRNA expression. Although not significant with a 5% level of significance, there is a tendency of increase in all isoforms with the highest relative elevation in VEGFA_120_. Statistical method used was Student t-test. (n_wt_ = 5, n_N-CAM+/−_ = 2, n_N-CAM−/−_ = 4). Scale bars are 100 µm (A, B) and 20 µm (C–H).

To analyze whether the reduced size and number of tufts could be explained by diminished endothelial cell proliferation, we performed isolectin and BrdU-labeling. Indeed, N-CAM deficient retinas exhibited a dramatic reduction in endothelial cell proliferation within tufts (wt = 3.8±0.3 and N-CAM^−/−^ = 1.8±0.3, p = 0.005), whereas the remaining vasculature was unaffected (Figure 4A–D). The proliferation rate was normal in N-CAM deficient retinas both in the larger vessels (wt = 2.3±0.6 and N-CAM^−/−^ = 3.2±0.8, p = 0.39) and in the capillary bed (wt = 12.5±1.6 and N-CAM^−/−^ = 16.7±2.1, p = 0.19). It can, however, not be ruled out that the proliferation of non-endothelial cells within the tufts and the retinal vasculature also could be affected by N-CAM deletion.

The blood vessel structure and sprouting of the revascularization front appeared similar between N-CAM deficient and control retinas (Figures 4E–F). The tufts formed by pathologic intravitreous neovascularization also display filopodia (Figure 4G), even though they appear shorter and point in all directions. No change in either kind of sprouting was observed in N-CAM deficient mice during OIR (Figure 4F, H).

To investigate whether the decrease in pathological neovascularization upon N-CAM deficiency was linked to *VEGFA* expression changes, *VEGFA* mRNA levels were quantified at P17 when *VEGFA* levels are the highest [20]. In contrast to what was expected, there was an increase in *VEGFA* mRNA expression, despite reduced endothelial cell proliferation within the retinal tufts (Figure 4A–D, I).

### Normal perivascular ECM distribution both in the developing retina and OIR tufts despite N-CAM ablation

**Figure 5:**
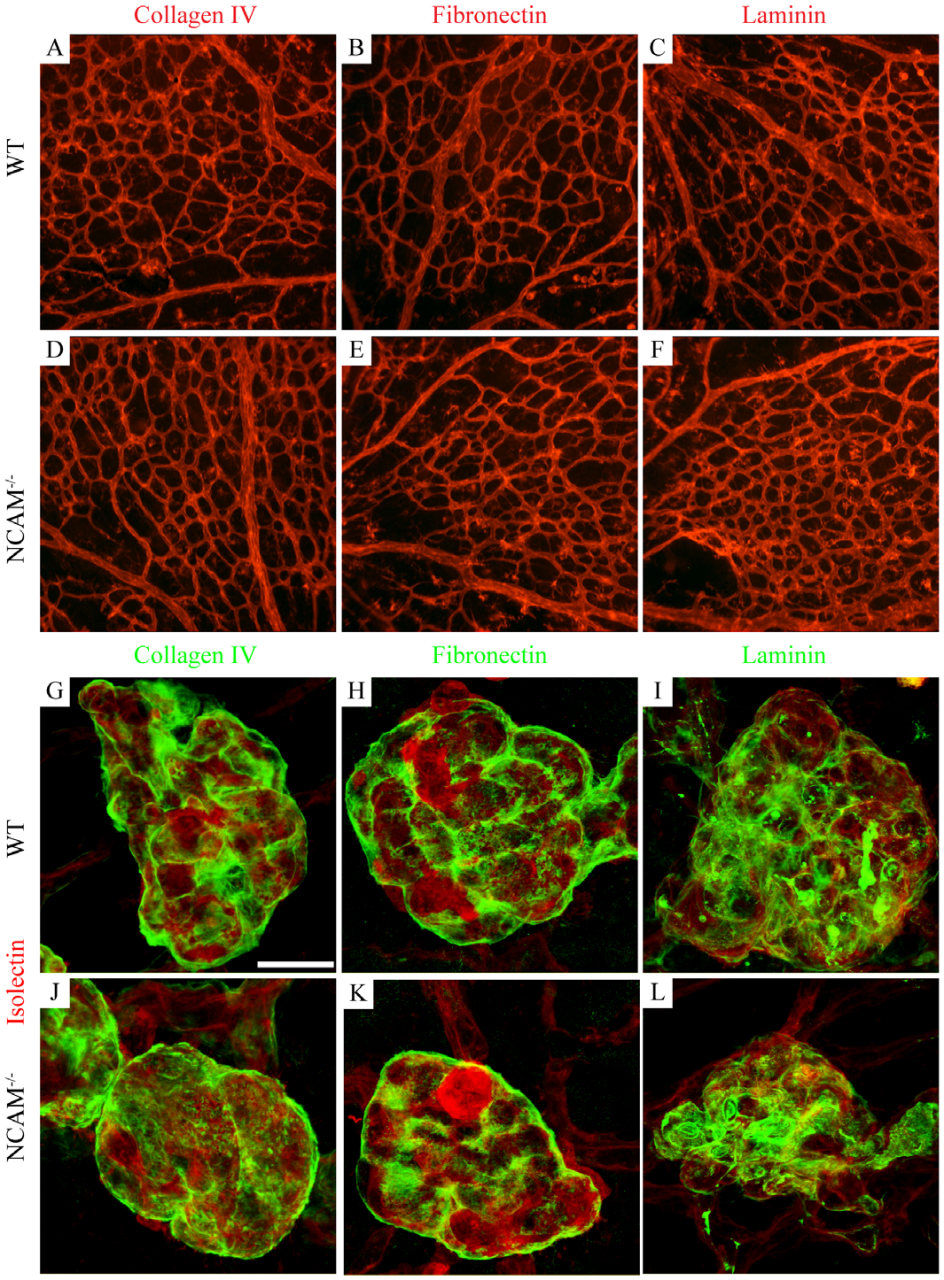
N-CAM deficiency does not affect the ECM distribution in the developing retina or in OIR tufts. (A–F) Overview images of retinas stained with antibodies against collagen IV (A, D, G, J), fibronectin (B, E, H, K) or laminin α1 (C, F, I, L) (red). Collagen IV, fibronectin and laminin α1 were expressed in the whole developing retinal vasculature at P5 (A–C). Expression analysis of ECM components after OIR revealed a strong expression of collagen IV (G, J), fibronectin (H, K) and laminin α1 (I, L) (green) around the epiretinal tufts, visualized by isolectin (red). No differences were observed in the N-CAM deficient animals (D–F, J–L). Scale bar is 20 µm.

To investigate whether N-CAM exhibited similar effects on the perivascular ECM deposition during pathological angiogenesis in the retina as during tumor angiogenesis, expression of the basement membrane proteins collagen IV, fibronectin and laminin α1/γ1 was studied. However, N-CAM ablation resulted in no change in the expression of these ECM molecules during normal blood vessel development (Figure 5A–F, and S3) or during OIR (Figure 5G–L). All ECM molecules tested (collagen IV, laminin α1/γ1 and fibronectin) were evenly distributed on the vasculature (Figure 5A–F and data not shown). Epiretinal tuft were to a great extent covered by collagen IV, fibronectin and laminin α1 (Figure 5G–L).

### N-CAM ablation affects TGF-β and FGFR mRNA expression in the retina

In an attempt to elucidate the mechanism behind the tuft specific decrease in proliferation rate, genes known to control endothelial cell proliferation, including FGF1, FGF2, TGF-β and IGF-1 together with genes known to be involved in signaling together with N-CAM, including FGFR and EGFR, were analyzed using quantitative PCR (QPCR) on OIR retinas.

**Figure 6:**
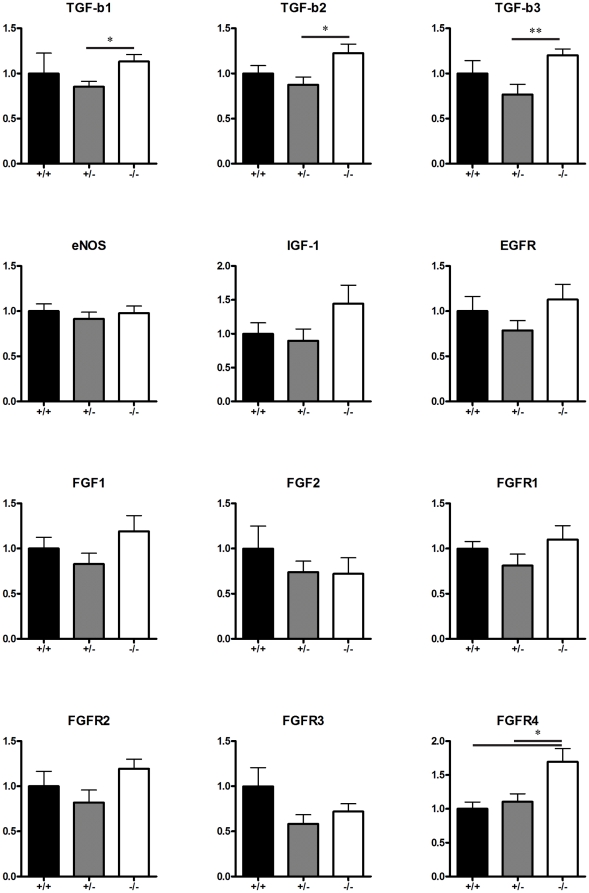
N-CAM affects TGF-β and FGFR4 expression levels in retinas during OIR. QPCR was used to quantify the expression of vascular related genes in retinas after OIR. All isoforms of TGF-β (n_wt_ = 6, n_N-CAM+/−_ = 8, n_N-CAM−/−_ = 7), were upregulated in N-CAM^−/−^, FGFR4 (n_wt_ = 6, n_N-CAM+/−_ = 8, n_N-CAM−/−_ = 7) was significant upregulated in the N-CAM^−/−^ mutants compared to the N-CAM^+/−^ and wt whereas the other genes were not statistically significantly altered, eNOS ( n_wt_ = 5, n_N-CAM+/−_ = 3, n_N-CAM−/−_ = 4), IGF-1, FGF1, FGF2, FGFR2, FGFR3and EGFR (n_wt_ = 6, n_N-CAM+/−_ = 8, n_N-CAM−/−_ = 7). Statistical method used was Student t-test. * = p<0.05.

Both FGF1 (aFGF) and FGF2 (bFGF) are known to stimulate endothelial cell proliferation [21], [22] but we did not detect any statistical significant altered mRNA levels in the N-CAM mutants (Figure 6). TGF-β is known to affect endothelial cells in various ways and have been implicated both to stimulate and to inhibit endothelial cell proliferation [23]. For all three isoforms we detected a statistical significant up regulation in N-CAM mutant retinas compared to the heterozygote's, whereas compared to the wt the up regulation did not reach significance (Figure 6).

IGF-1 signaling has previously been shown to stimulate endothelial cell proliferation in OIR [24], [25] but we could not observe any statistically significant mRNA expression changes in the N-CAM mutants (Figure 6). Both the EGFR and the FGFRs have been shown to functionally interact with N-CAM [26], [27]. FGFR4 was statistically significant upregulated in the N-CAM^−/−^ mutants compared to the N-CAM^+/−^ and wt. The expression level of EGFR was not altered by N-CAM (Figure 6).

## Discussion

In OIR, hyperoxia primarily results in blood vessel regression. Returning to normoxia creates a hypoxic milieu in the avascular area, which stimulates revascularization and pathologic neovascularization. The normal direction of retinal blood vessel infiltration due to hypoxia is from the superficial layer down through the deeper layer of the retina. However, it is not known why the vessels penetrate the exceeding inner limiting membrane to form epiretinal tufts under pathological conditions. Although the tufts appear malfunctional, they provide the underlying retina with physiological oxygen levels, which presumably lower VEGFA expression by the astrocytes [28].

In the present study we show that N-CAM ablation does not affect the normal developmental retinal angiogenesis or the revascularization of the retina after OIR but decreases, in a gene-dosage dependent way, the epiretinal tuft formation.

During normal retinal development N-CAM is expressed in a pattern suggesting that astrocytes are the cell of origin close to the vasculature. After OIR N-CAM is accumulated around the epiretinal tufts, also indicating astrocytic origin. Even though astrocytes seem to be the probable source of N-CAM we cannot rule out that other cell types, as Müller cell and microglia, also express N-CAM.

In agreement with our observations that the revascularization was unaffected whereas the tuft formation was decreased, the reduced endothelial proliferation rate was restricted to the tufts.

The migrating endothelial front uses tip cell filopodia extensions to guide their movement by following the pre-existing VEGFA expressing astrocyte network [9]. Deficient sprout establishment could potentially lead to reduced endothelial cell migration and proliferation [9]. In the N-CAM deficient retinas, tip cell sprouting was unaffected both during normal development and OIR. In OIR the epiretinal tufts also produce short and randomly directed sprouts of which neither was affected in the N-CAM deficient retinas.

A VEGFA gradient has been shown to be crucial for endothelial migration and proliferation in the retina [9], [29]. Whereas VEGFA is essential for developmental angiogenesis within the retina [4], it has also been associated with pathologic angiogenesis in ocular diseases [30], [31], [32], [33]. VEGFA is encoded by one gene, which after alternative splicing generates at least three major isoforms; VEGFA_188_, VEGFA_164_ and VEGFA_120_ [34]. Guidance of tip cell filopodia is dependent on the correct relationship between the expression levels of all three isoforms. Therefore, disturbed expression of any of the isoforms may cause defective filopodia guidance, endothelial migration and proliferation [9]. It was therefore of interest to analyze the effect of N-CAM ablation on the expression level of each isoform. In agreement with previous studies, the 164 isoform was the most abundant, followed by 120 and 188 (data not shown) [35]. Statistical analysis failed to reveal significant differences in VEGFA isoform expression between N-CAM mutant and wt retinas in OIR, suggesting that the reduced EC proliferation is not due to altered VEGFA levels. N-CAM may thus regulate endothelial cell proliferation by VEGFA-independent pathways. One such pathway is endothelial nitric oxide synthase (eNOS)-mediated signaling [36]. Nitric oxide (NO) is a free radical produced by NOS of which there are three variants. eNOS, predominantly expressed in the plasma membrane of vascular endothelial cells [37], [38], has been implicated to play a proangiogenic role [39], [40]. Mice deficient in eNOS express an OIR phenotype, which is very similar to the one presented in this work, with less pathologic angiogenesis and elevated VEGFA levels [36]. To investigate whether eNOS was transcriptionally regulated in N-CAM deficient OIR retinas we measured eNOS mRNA expression levels. No difference in eNOS mRNA expression was detected between N-CAM mutant and wt retinas. To further elucidate the mechanism behind the reduced proliferation in the vascular tufts, the expression of genes known to play a role in endothelial cell proliferation or N-CAM signaling were analyzed by QPCR on OIR retinas. Among the genes analyzed, all three isoforms of TGF-β and FGFR4 were up regulated in N-CAM^−/−^. However, since N-CAM only affects pathological angiogenesis, RNA extraction from whole retina might not be sensitive enough to pick up changes in mRNA expression only from the pathological vasculature. Both TGF-β and FGFR4 could play a role in the mechanism by which N-CAM regulates tuft formation during OIR.

N-CAM deficiency may also directly or indirectly affect the distribution of growth factors bound to the ECM. It has previously been shown that if the basement membrane of blood vessel is injured or degraded, e.g. by defective ECM deposition, the underlying endothelial and mural cells get intimately exposed to surrounding growth factors and respond by starting to proliferate [41], [42]. Consistently, ECM molecules have been implicated in endothelial cell proliferation during angiogenesis [43]. We previously showed that N-CAM-deficiency during tumor progression resulted in diminished expression of ECM components and defective perivascular ECM deposition [19]. Further, N-CAM has been shown to inhibit expression of matrix degrading enzymes (matrix metalloproteinases, MMPs) [44] which could indicate a proliferation promoting property. In this study, the proliferation in the pathological retinal tufts was down regulated in N-CAM-deficient mice which rather would expect ECM-inhibiting or MMP-promoting properties of N-CAM. We found no evidence that N-CAM deficiency has any effect on ECM components in the retina during normal development or OIR. It can, however, not be ruled out that other ECM components than the ones examined in this study might be affected in the N-CAM mutants. Further studies are needed to reveal whether there are mechanistically differences in N-CAM's role during OIR and tumor angiogenesis.

In conclusion, we show that N-CAM is involved in hypoxia regulated endothelial cell proliferation *in vivo*. Hypoxia-exposure in the OIR model resulted in a gene-dosage dependent decrease in pathological angiogenesis, as revealed by reduced formation of epiretinal tufts, in N-CAM heterozygous and homozygous mice, whereas normal retinal vasculature development appeared unaffected. These observations are consistent with our recent finding that N-CAM deletion limits pathological angiogenesis during tumor progression [19]. Altogether, these findings emphasize a novel role of N-CAM in pathological angiogenesis.

However, the underlying mechanism for N-CAM's role in pathological angiogenesis during tumorigenesis and OIR appears to differ. Whereas N-CAM maintains vessel integrity by promoting pericyte-endothelial cell-cell interactions during tumor angiogenesis, N-CAM is required for endothelial cell proliferation during formation of the epiretinal tufts. The distinct accumulation of N-CAM around the epiretinal tufts suggests a potential role in their formation during OIR. Indeed, N-CAM ablation resulted in a decrease in endothelial cell proliferation specifically within tufts, which presumably explains the reduction in tuft formation. This finding would be consistent with reduced expression of the major proliferation growth factor VEGFA. However, instead, we found that VEGFA mRNA expression was increased in N-CAM^−/−^ mice relative to the controls. The increase in VEGFA levels could either be a compensatory effect of the defective proliferative response, or a consequence of reduced formation of epiretinal tufts, which normally lower VEGFA expression in astrocytes after providing the underlying retina with higher oxygen levels [28].

By screening several genes involved in endothelial cell proliferation or N-CAM signaling we identified TGF-β and FGFR4 to be potential mechanistically targets of N-CAM in vascular tuft formation.

Based on our findings, we propose that N-CAM may be a potential target of clinical relevance for diseases caused by proliferative retinopathy in humans.

## Materials and Methods

### Ethical statement/Mice

N-CAM^+/−^ (with C57/BL6 background) [45] mice were bred to generate wt, N-CAM^+/−^ and N-CAM^−/−^ littermates. They were housed and bred in accordance with regulations for the protection of laboratory animals, after approval from the local ethical committee. The animals were housed on a 12∶12-hour light–dark cycle and food and water were available *ad libitum*.

### Tissues

*Oxygen induced retinopathy (OIR)*; Mice at postnatal day 7 (P7) were exposed to 75% oxygen for 5 days together with their mothers in an oxygen chamber [3], the oxygen concentration was checked daily. The mice were then transferred back to normal air (21% oxygen) for 5 days after which the mice were sacrificed and the eyes were dissected for whole mount immunostainings.

*Others*; Mice at P5, P7 and P10, respectively, were sacrificed and eyes were dissected for immunostainings.

The FITC dextran perfusions were carried out as previously reported [46].

### Immunohistochemistry

Retinas were prepared and stained according to earlier published protocol [9]. Shortly, the mice were sacrificed and the eyes collected and placed in PBS on ice. After a short (2 minutes) fixation in 4% PFA the retina was dissected. To avoid damage to the epiretinal tufts during this procedure the hyaloid vessels were gently removed. The retina was then fixed for an additional 2 hours. The blood vessels were visualized by staining with biotinylated isolectin B4 (*Bandeiraea simplicifolia*) (L-2140; Sigma-Aldrich). Antibodies used were; rabbit anti-N-CAM (1∶100 dilution; a gift from Dr. E. Bock), rat ant-N-CAM (1∶100; Abcam), goat-anti PECAM (1∶500; R&D), FITC-conjugated rat anti-BrdU (1∶200; Nordic Biosite), rabbit anti-GFAP (1∶100; Sigma), rabbit anti-collagen IV (1∶250; Biogenesis), rabbit anti-fibronectin (1∶200; DAKO), mouse monoclonal anti-laminin α1 (generous gift from Dr. P. Ekblom), rabbit anti-laminin γ1 (generous gift from Dr. M. Durbeej-Hjalt) rabbit anti-NG2 (1∶200; Chemicon). Conjugated secondary antibodies from Molecular Probes and Jackson ImmunoResearch Laboratories were used. Flat mounted retinas or sections were analyzed by fluorescence microscopy using a Nikon E1000 or a Zeiss Axioplan 2 microscope and by confocal laser scanning microscopy using a Leica LCS NT or Zeiss LSM 780. Images were processed using Adobe Photoshop® and or Imaris.

### QPCR

Retinas from mice exposed to the OIR treatment were dissected in ice cold PBS and total RNA was prepared using the RNeasy mini kit (Qiagen) with the RNase free DNase (Qiagen) treatment according to the manufacturer's instructions. Reverse transcription (iScript, BioRad) and QPCR assays using SYBR Green I as detection chemistry was performed as described [47]. Primer sequences are shown in Table S1. PCR products were checked by agarose gel electrophoresis and melting curve analysis. Expression data were normalized against Tubb5. The expression level of VEGFA_188_ was found to be significantly lower (<10 fold) than the expression of VEGFA_188+164_, consequently VEGFA_188+164_∼VEGFA_164_. The ratio between VEGFA_188+164_ and VEGFA_188+164+120_ (∼VEGFA_164_ ∶ VEGFA_164+120_) gives the relative difference in expression level between the VEGFA_164_ and VEGFA_120_ isoforms.

### Blood vessel area quantification

Pictures of whole mount retinas after OIR were taken at 200×. For measurement of tuft-, avascular-, and total retina-area the Velocity software was used, and by modulating intensity settings the different areas could be quantified. By letting the software identify only the parts of the vasculature with highest intensity of the isolectin staining, the tufts were marked and the area for each tuft was quantified. By letting the software identify the area with any staining at all, including the very low background, the total retina was marked and the area was quantified. By setting the intensity level very low, the avascular part in center of the retina was marked, and the area was quantified.

### Blood vessel diameter measurement

Pictures of P10 whole mount isolectin stained retinas were taken att 200×. The vessel diameter (measured in the computer software Photoshop CS) was measured on 30 blood vessels of the capillary network in 3 wt and N-CAM^−/−^ mice. The data is presented in length units as average ±SEM.

### ECM quantification

P5 retina samples were stained for PECAM and fibronectin or collagen IV or laminin γ1. One field/retina (20× objective) in the capillary bed was scanned using Zeiss LSM 780 confocal.

Using Imaris the blood vessel volumes (PECAM) and the ECM volumes were quantified. The ECM volume/blood vessel volume were compared between wt and N-CAM^−/−^. The data was normalized to wt and presented as average ±SEM. n_all groups_ = 5.

### Quantification of endothelial cell proliferation retinas after OIR

Pictures of whole mount retinas after OIR were taken at 200×.

#### Tufts

BrdU labeled cells were counted in 338 tufts from 3 wild type retinas and 123 tufts from N-CAM^−/−^ retinas. The area of each tuft was measured with the same technique as mention above in the section “blood vessel quantification”. The number of BrdU labeled cells per tuft area unit was calculated and the average was calculated for each retina. Student t-test was performed on n = 3. The data is presented as average ±SEM BrdU-labeled cells/area unit.

#### Capillaries

BrdU labeled cells were counted in 34 fields of the capillary network of 3 wt retinas and 29 fields of the capillary network of N-CAM^−/−^retinas. The area was measured in Photoshop and the number of BrdU labeled cells per area unit was calculated. The average was calculated for each retina and Student t-test was performed on n = 3. The data is presented as average ±SEM BrdU-labeled cells/area unit.

#### Large vessels

BrdU labeled cells were counted in 24 large vessels of 3 wt retinas and 28 large vessels of N-CAM^−/−^retinas. The length of each vessel was measured in Photoshop and the number of BrdU labeled cells per length unit was calculated. The average was calculated for each retina and Student t-test was performed on n = 3. The data is presented as average ±SEM BrdU-labeled cells/length unit.

## Supporting Information

Figure S1 **N-CAM is expressed in the mouse retinal blood vessel layers and co-localize with astrocytes and N-CAM ablation does not affect blood vessel leakage in retinas after OIR.** (A) Retinal sections from wt P7 were stained for N-CAM (green), PECAM (blue) and GFAP (red). N-CAM was expressed in the blood vessel layer and co-localize with GFAP expressing astrocytes but is also expressed in deeper retinal layers. Astrocytes enter the retina from the optic nerve (on) and first form a superficial plexus which is seen as the GFAP^+^ rim close to the vitreous body. As insets high magnification optical sections are shown. Scale bar = 100 µm. (B) FITC-dextran (green) perfusion of retinas after OIR revealed almost no leakage of the retinal vasculature and there was no difference between N-CAM^−/−^ and wt. Isolectin staining (red), FITC-labeled dextran (green).
(TIF)

Figure S2 **N-CAM ablation decreases the number of tufts.** (A) The total number of tufts per retina was 2.8 times higher in WT compared to NCAM^−/−^ (Student t-test, p<0.05, n = 3 for WT and NCAM^−/−^). (B) The tuft areas were lognormally distributed in both WT and NCAM−/− and the number of tufts was lower for NCAM^−/−^ than WT for all sizes of area. (Tuft areas were pooled for 3 WT and 3 NCAM^−/−^ mice, respectively).
(TIF)

Figure S3 **ECM volume quantification.** The blood vessel volumes (PECAM) and ECM (fibronectin, collagen IV and laminin γ1 respectively) volumes were quantified in p5 retina samples using confocal microscopy and Imaris software. The ECM volume/blood vessel volume were compared between wt and N-CAM deficient retinas, no significant difference could be detected, collagen IV p = 0,8026, fibronectin p = 0,2705 and laminin γ1 p = 0,8002.
(TIF)

Table S1 **QPCR primer sequences.**
(DOCX)
