# Supplementary figures and images for: N-CAM Exhibits a Regulatory Function in Pathological Angiogenesis in Oxygen Induced Retinopathy
Source: PLoS One. 2011 Oct 17;6(10):e26026. doi: 10.1371/journal.pone.0026026 (PMC3197149; doi:10.1371/journal.pone.0026026)

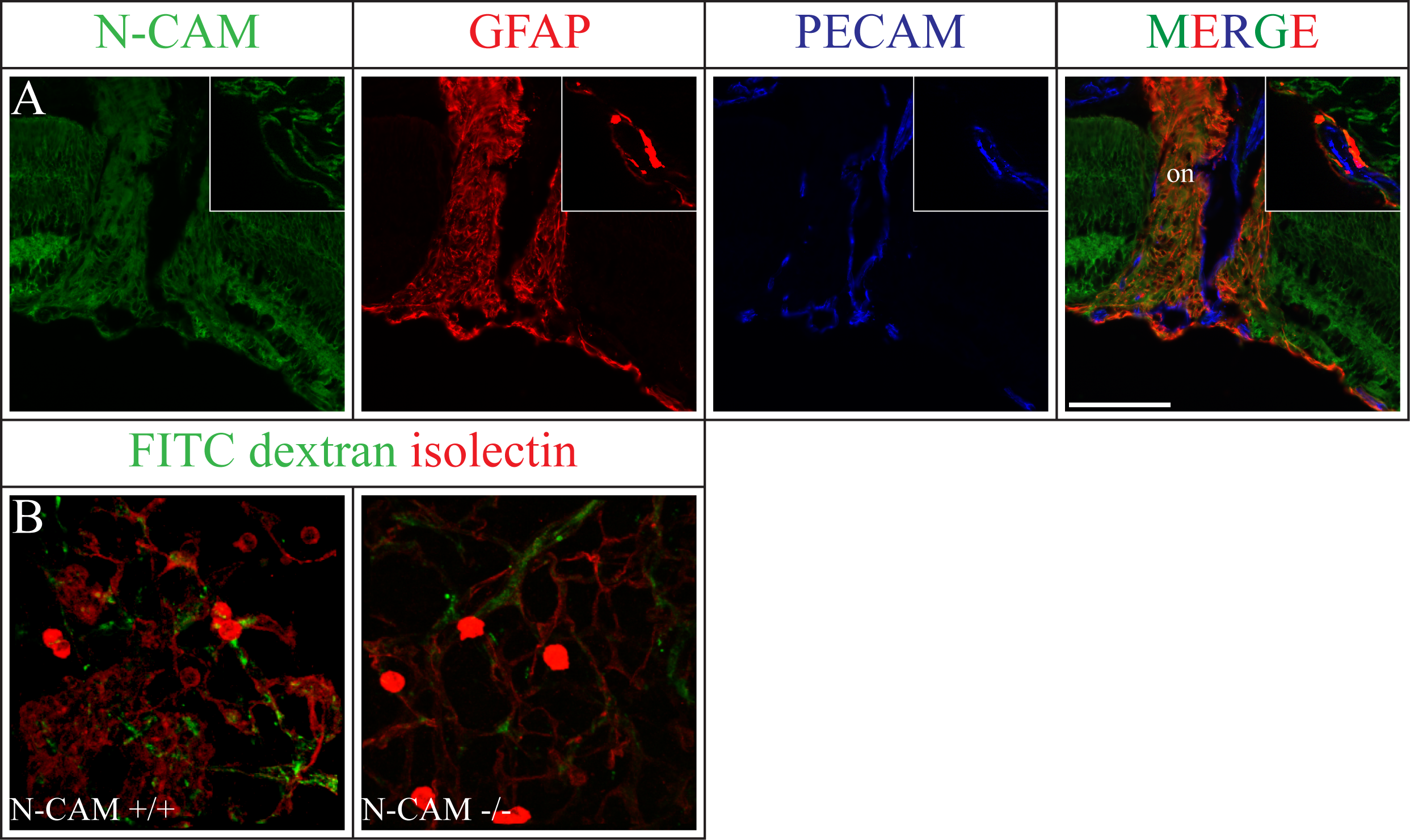

Supplement: Figure S1 — N-CAM is expressed in the mouse retinal blood vessel layers and co-localize with astrocytes and N-CAM ablation does not affect blood vessel leakage in retinas after OIR. (A) Retinal sections from wt P7 were stained for N-CAM (green), PECAM (blue) and GFAP (red). N-CAM was expressed in the blood vessel layer and co-localize with GFAP expressing astrocytes but is also expressed in deeper retinal layers. Astrocytes enter the retina from the optic nerve (on) and first form a superficial plexus which is seen as the GFAP+ rim close to the vitreous body. As insets high magnification optical sections are shown. Scale bar = 100 µm. (B) FITC-dextran (green) perfusion of retinas after OIR revealed almost no leakage of the retinal vasculature and there was no difference between N-CAM−/− and wt. Isolectin staining (red), FITC-labeled dextran (green). (TIF) [file pone.0026026.s001.tif]

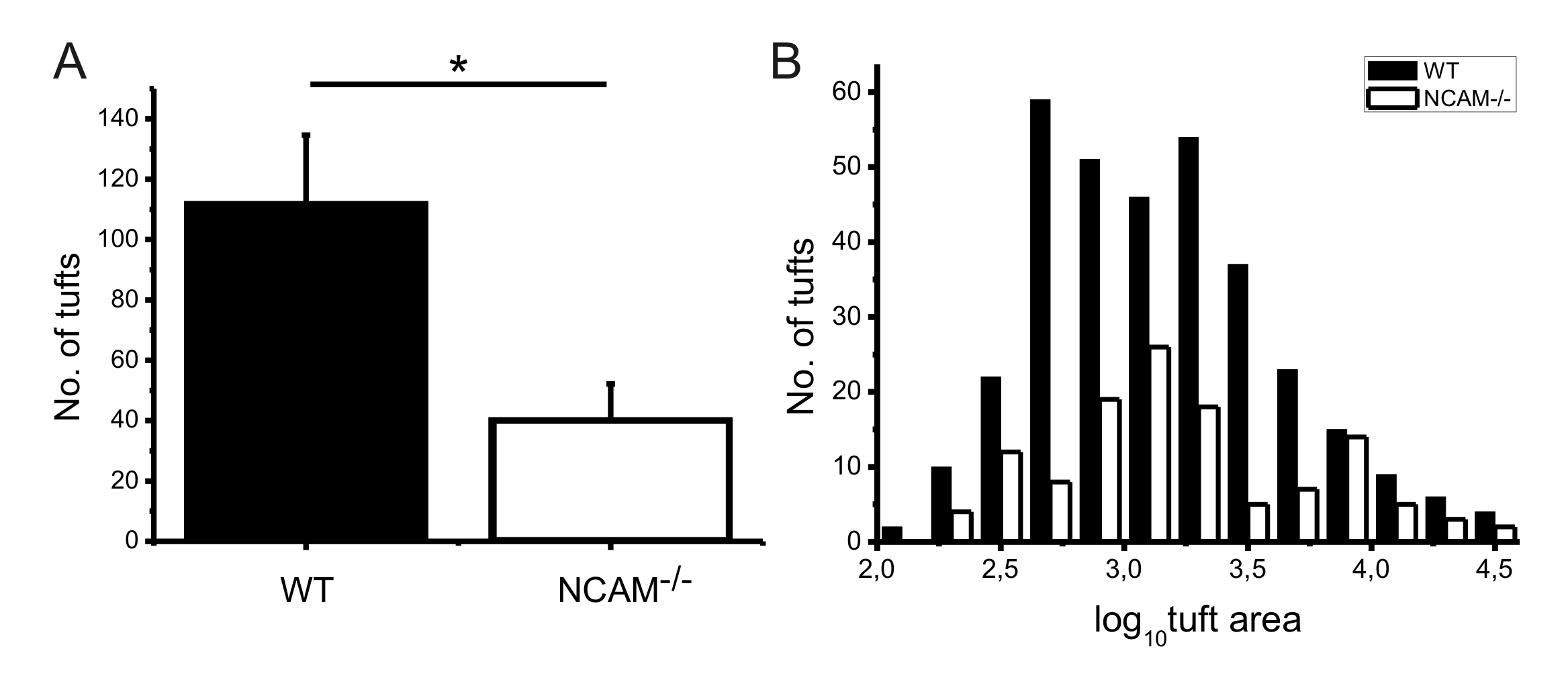

Supplement: Figure S2 — N-CAM ablation decreases the number of tufts. (A) The total number of tufts per retina was 2.8 times higher in WT compared to NCAM−/− (Student t-test, p<0.05, n = 3 for WT and NCAM−/−). (B) The tuft areas were lognormally distributed in both WT and NCAM−/− and the number of tufts was lower for NCAM−/− than WT for all sizes of area. (Tuft areas were pooled for 3 WT and 3 NCAM−/− mice, respectively). (TIF) [file pone.0026026.s002.tif]

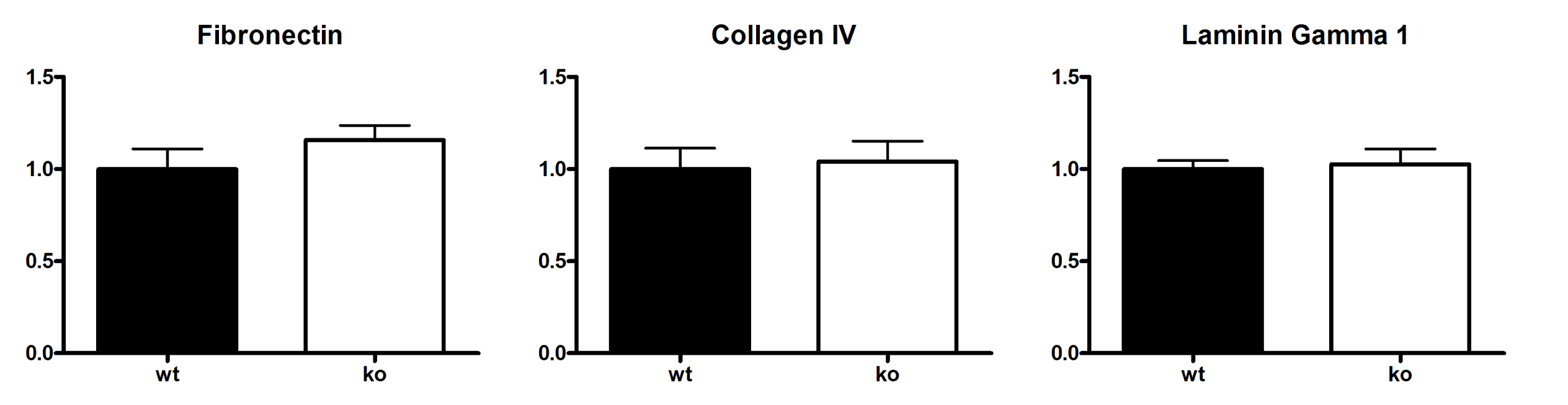

Supplement: Figure S3 — ECM volume quantification. The blood vessel volumes (PECAM) and ECM (fibronectin, collagen IV and laminin γ1 respectively) volumes were quantified in p5 retina samples using confocal microscopy and Imaris software. The ECM volume/blood vessel volume were compared between wt and N-CAM deficient retinas, no significant difference could be detected, collagen IV p = 0,8026, fibronectin p = 0,2705 and laminin γ1 p = 0,8002. (TIF) [file pone.0026026.s003.tif]
