# Supplementary material for: N-CAM Exhibits a Regulatory Function in Pathological Angiogenesis in Oxygen Induced Retinopathy
Source: PLoS One. 2011 Oct 17;6(10):e26026. doi: 10.1371/journal.pone.0026026 (PMC3197149; doi:10.1371/journal.pone.0026026)
Supplement: Table S1 — QPCR primer sequences. (DOCX) [file pone.0026026.s004.docx]

**Table S1. QPCR primer sequences**

Gene Fwd primer (5’-3’) Rev primer (5’-3’)

Tubb5 CCTTCATTGGAAACAGCACA CCTCCTCTCCGAAATCCTCT

VEGFA_188_ AGGAAAGGGAAAGGGTCAAA TCACATCTGCAAGTACGTTCG

VEGFA_188+164_ CCTTGTTCAGAGCGGAGAAA TTAATCGGTCTTTCCGGTGA

VEGFA_188+164+120_ CACGACAGAAGGAGAGCAGA ATCAGCGGCACACAGGAC

Tgfb1 CAATTCCTGGCGTTACCTTG AGCCCTGTATTCCGTCTCCT

Tgfb2 CATCCCGCCCACTTTCTAC CCTCCGCTCTGGTTTTCAC

Tgfb3 CGAGTGGCTGTTGAGGAGA CAGGTGTGGGTTGTGGTG

Igf1 CTCTGCTTGCTCACCTTCAC CACTCATCCACAATGCCTGT

Fgfr1 ATCCGCAGCCTCACATTC TAGAGTTACCCGCCAAGCAC

Fgfr2 TACCCTCACAGAGACCCACA GAATCGTCCCCTGAAGAACA

Fgfr3 CGGAGCGAATGGATAAGAAA AGGAGATGGAGGGGGTAGG

Fgfr4 GACCGAGGATGATGTGATGA CGCAGCAGTGAGAAGAGC

Fgf1 GGAAACTACAAAAAGCCCAAACT TGGTGTCTGCGAGCCGTAT

Fgf2 GCTATGAAGGAAGATGGACGG AGACATTGGAAGAAACAGTATGGC

Egfr TACCTATGGATGTGCTGGGC AGGGGAGTCAGAGATGGCTT

Nos3/Enos GCAGGCTCTCACCTACTTCC GCTGTTCGCTGGACTCTTCT
